# Errors, Mistakes, and Failures, Old and New

**Published:** 1905-10

**Authors:** C. W. Strang

**Affiliations:** Bridgeport, Conn.


### ERRORS, MISTAKES, AND FAILURES, OLD AND NEW.[1]

BY DR. C. W. STRANG, BRIDGEPORT, CONN.

Mr. President, and Gentlemen of the Institute of Stomatology,—In casting about in my own mind for a subject for this occasion, of some that suggested themselves were "Successes and Achievements," and "Errors, Mistakes, and Failures." I have selected the latter, believing it will prove more interesting to my audience, inasmuch as it is the successes and achievements that we hear of or are most frequently recorded in our dental publications.

I congratulate myself that the subject is a broad one; the material is genuine, for it has been personally made, and whatever may be said, I shall hardly be wandering from the text.

While it may be true that too much brooding over one's failures is depressing to mind and body and productive of harm, yet most of us have learned from experience and observation that failures many times have been blessings in disguise and the stepping-stones to success and renown.

---

[1] Read before The New York Institute of Stomatology, June 6, 1905.

The words error, blunder, mistake, and failure, while sometimes used interchangeably, are not synonymous, but in the order named stand somewhat in the relation of sequence, the one leading up to the other.

Recalling my advent as a dental practitioner, without any training along the lines of practical business, the unsuitable light for operative purposes had in the first two offices occupied, the scanty equipment, both at the chair and in the laboratory, my heart goes out in sympathy to any struggling young man who is similarly situated; and the wonder is that failures are not more numerous and success less frequent.

In our early days it was strenuously advocated by some eminent in the profession that if teeth are worth filling at all, they are worth filling with gold.

I absorbed enough of that sentiment to warrant me in now declaring that the experience gained in the manipulation of gold was purchased at a high rate, in so far as the interests of our patients were concerned.

Cohesive gold fillings inserted in the anterior teeth of children twelve or fifteen years of age, no rubber dam, no obtunder, a wooden wedge crowded or driven between the teeth against the gum—the thought of the anguish then inflicted makes us shudder now.

It was a mistake, for in a year or two I had the great mortification of seeing much of that work failing at the cervical border, and had it not been for the operations made in the masticating surfaces of the molars and which generally did fairly well, I would have been left without a reputation, or possibly with a bad one.

Prior to the introduction of the rubber dam the large percentage of failures arising from the impracticability of properly treating and filling the root-canals of posterior teeth, particularly the inferior molars, stimulated experiment along the line of conservative pulp-treatment. Of all the agents employed for capping exposed pulps, oxychloride of zinc received the greatest boom.

We can recall as though it were but yesterday hearing Dr. Atkinson tell how the operation should be made. It ran something like this: Remove the decay from the cavity, prepare cavity as indicated for filling to be inserted, thoroughly dry out the cavity, sop the exposed pulp with creosote,—the creosote unites with the albumin and forms an insoluble compound. That insoluble compound, what hopes we built upon it! Mix the oxychloride to the consistency of cream, place into the cavity, and gently coax it over the pulp. After it has had time to harden put in the filling. Kiss your patient good-bye and all will be well. I took up pulp-capping and tried faithfully to follow Dr. Atkinson's directions, cutting out the kissing part. Josh Billings said, when he heard them sing, "Come where my love lies dreaming," he never went because he did not think it would be right.

What were the results? Just what we would now expect where little or no thought is given to pathological conditions. A few thus treated remained comfortable and retained vitality for longer or shorter periods; but many became so painful that relief was sought in extraction or other treatment. In some instances, where actual exposure had been repaired by a deposit of secondary dentine and conditions promised well, congestion set in, and it became necessary to devitalize the pulp and treat accordingly.

It was the failures of long years ago to treat successfully or give much relief in those cases, where pyorrhœa alveolaris was getting in its work, that gave a stimulus to be thorough in the care of the mouths of our patients, systematically and regularly removing the foreign deposit, believing that under those conditions the dreaded disease would not be as likely to develop.

I have been abundantly rewarded for the time and labor expended in this particular kind of work, though it has not been as fascinating as making gold operations, and not as remunerative.

Personal experience with an exposed pulp in each of the inferior sixth-year molars in my early teens afforded an opportunity to obtain knowledge concerning that malcondition that certainly could not have been procured in any other way, and I also retain pronounced recollections of agonies endured from an occasional accidental impacting of some foreign substance upon a congested pulp.

An alveolar abscess at the root of each of these molars for ten or twelve years afforded an opportunity for the acquisition of more valuable information.

The possibilities lurking in pulpless teeth made such a profound impression upon me that I was stimulated in after years in our examinations to be scrupulously careful not to overlook or fail to locate cavities in obscure places, avoiding as much as possible the necessity of devitalizing pulps.

With the introduction of the dental engine and its equipment, many of the instruments employed in cavity preparation prior to that time have gradually become obsolete, and presumably there are few dental operators who have come into the profession during the last score of years whose instrument cabinet contains a good assortment of excavators, chisels, hand cavity burs, and plug finishing burs; and this we regard as a mistake, and as a result of the mistake more pain is inflicted, frail walls are unwittingly left to be broken down at a later time, necessitating a repetition of the operation of filling.

A few well-directed strokes of the enamel chisel in the hand of the skilled operator will, in a moment or two, effectually remove that which should be sacrificed, and with the infliction of less pain and discomfort to the patient than that produced by abrasive wheels and burs revolved rapidly in the hand-piece of the dental engine.

Again, with the advent of the dental engine the final polishing of the teeth with stick and pumice-stone was very generally abandoned by most operators, and the rubber cups and points substituted for that purpose, and this, in the light of present developments, we believe has been a mistake, and my convictions are deepened and strengthened by almost daily experience and observation.

A recent case in practice, which with your permission I will cite, has a bearing upon this point; and although medicaments were also employed in the treatment of this case, the chief factor in bringing about a normal and healthy condition of the mouth was absolute cleanliness of the teeth obtained by the frequent and thorough use of the orange-wood points carried in the porte-polishers and loaded with pumice-stone.

The case is as follows: In the afternoon of the last Sunday of October a gentleman, by direction of his family physician, called upon me and requested me to treat his wife, who was suffering with a badly inflamed mouth. The case, he stated, had been treated from time to time by a dentist for about three months; but, as it grew worse rather than better, had been transferred to an oral surgeon, who, after a few days' treatment by spraying the mouth, advised the patient to consult a physician, that she might also receive systemic treatment. Under these circumstances the patient decided to return to her home for treatment.

An examination revealed the following condition: The lips and a space extending for a half-inch from the border of the mouth was a solid mass of ulcers resembling very much the fever sores that are commonly seen about the mouths of children. Around all the molars, both inferior and superior, the gums were spongy and puffed and rolled over upon the masticating surfaces of some of them, and hemorrhage was induced by slight interference. The tongue, roof of the mouth, and, indeed, the mucous membrane of the mouth everywhere was thickly studded with ulcer patches. Articulation was so interfered with by the excessive inflammation that it was almost impossible to understand the patient in conversation. The treatment consisted, first, of an application of rose water and glycerin to the lips and surrounding parts, to render them as pliable as possible, then the removal of whatever of hard deposit that was attached to the teeth, and this was not abundant; then about two hours was spent in polishing the teeth with the orange-wood points loaded with pumice-stone moistened with phenol sodique. As each tooth was polished the gum tissue about it was syringed with tepid water.

The patient was directed to rinse the mouth every hour with Phillips's milk of magnesia, every other day alternating with precipitated chalk, 2 parts, carbonate of magnesia, 1 part, mixed to the consistency of cream, a tablespoonful to a glass of water. At the end of five days the case was again seen. No perceptible improvement was observed. The polishing and cleansing was repeated, and the antacid treatment continued. At the end of another period of five days the case was again seen, and the improvement was remarkable.

The treatment as described was continued for one month, at intervals of five or six days, when the patient was dismissed with the mouth in a perfectly normal condition.

In conclusion, I desire at this time to tender to Dr. D. D. Smith, of Philadelphia, acknowledgments for courtesies extended to me nearly two years ago at his office. Also to say that through his teachings came a preparation that has enabled me to treat so successfully not only the case just referred to, but others of a serious, if not similar nature.

About seven years ago a lady, who had been a patient of mine for twenty-five years, consulted me with regard to a pain that she had in the left side of the lower jaw, beginning at about the median line and extending back to the ramus of the jaw. This disturbance had gone on for five or six weeks before she came to see me. There was over the ramus of the jaw a little swelling. The second bicuspid and the first molar had been removed a number of years before, and the lady was wearing a partial plate. The second molar had been badly worn away. I diagnosed the case as probably a dead pulp in this molar. Upon entering the tooth, I found the pulp dead in the anterior but alive in the posterior root. I devitalized the pulp in the posterior root, but to my surprise I could not find any opening into the anterior root; it seemed to have closed. After treating the tooth for a while I filled it temporarily. The pain continued. There was no change, especially in so far as the prominence on the face was concerned. I opened into the soft tissues, but found no pus, only a slight discharge of serum. I said to my clerk, "We have a malignant case here." I wrote up the history of the case and advised the patient to consult a specialist in this city. She consulted with him, with the result that he advised certain treatment, stating that he found nothing of malignancy in the case. She went under the care of her family physician, and I saw nothing of her for a week, when the physician came to me saying that there was pus somewhere in that region, and that if I would open into the pus cavity he would stand back of me. I did not care to go on with the case without having something more definite, so I telegraphed the specialist the opinion of the family physician. He advised a free incision. I met the physician at the home of the patient and made the incision. We found no pus there. The physician said there would be pus in three or four days. The wound was packed with iodoform gauze. On the following day the patient's face was badly swollen, but no pus had made its appearance. I insisted upon getting the advice of another specialist in this city, who also said that he saw nothing of malignancy. He believed it was a case of necrosis, and advised immediate operation. The patient came to this city and was operated upon. The molar tooth was removed. That tooth which was very firm at first, too firm I might say, at the time of the operation had no bone about it. It was removed without using any force whatever. Considerable disorganized bone was removed at that time. The patient remained in New York under treatment. In about six weeks I received a letter from the surgeon stating that he would again operate, and invited me to be present. I could not leave. A specimen was prepared at this time for microscopical examination, and the case was found to be malignant. The patient returned to her home about the holidays and lived until the following April. Somebody made a mistake in this case.

In June last a young man called upon me at the request of his physician. There was a growth under his tongue extending back as far as the second bicuspid on either side. It had been increasing for about a year, and had been very annoying for the last few months. I think if that case had come to me three years before, about the first thing I would have done would have been to put a knife into it. I know now that if I had done this I would have made a mistake. There was much deposit on the lower teeth. The teeth were thoroughly cleansed. In two weeks there was a marked improvement in the case. In about a month the mouth had returned to a normal condition.

